# Activated brown adipose tissue releases exosomes containing mitochondrial methylene tetrahydrofolate dehydrogenase (NADP dependent) 1-like protein (MTHFD1L)

**DOI:** 10.1042/BSR20212543

**Published:** 2022-05-20

**Authors:** Melvin Khee-Shing Leow, Anantharaj Rengaraj, Kothandaraman Narasimhan, Sanjay K. Verma, Jadegoud Yaligar, Giang Le Thi Thu, Lijuan Sun, Hui Jen Goh, Priya Govindharajulu, Suresh Anand Sadananthan, Navin Michael, Wei Meng, Xavier Gallart-Palau, Lei Sun, Neerja Karnani, Newman Siu Kwan Sze, S. Sendhil Velan

**Affiliations:** 1 Singapore Institute for Clinical Sciences, Agency for Science, Technology and Research (A*STAR), Singapore; 2 Lee Kong Chian School of Medicine, Nanyang Technological University (NTU), Singapore; 3 Cardiovascular and Metabolic Disorders Program, Duke-NUS Medical School, Singapore; 4 Yong Loo Lin School of Medicine, National University of Singapore (NUS), Singapore; 5 Department of Endocrinology, Tan Tock Seng Hospital (TTSH), 11 Jalan Tan Tock Seng, Singapore 308433, Singapore; 6 Laboratory of Molecular Imaging, Singapore Bioimaging Consortium, Agency for Science Technology and Research (A*STAR), Singapore; 7 Singapore Institute of Food and Biotechnology Innovation (SIFBI), Agency for Science Technology and Research (A*STAR), Singapore; 8 School of Biological Sciences, Nanyang Technological University, 60 Nanyang Drive, Singapore 637551, Singapore; 9 Hospital Universitari Institut Pere Mata, Institut Investigació Sanitària Pere Virgili (IISPV), Reus, Tarragona, Spain; 10 Bioinformatics Institute, Agency for Science, Technology and Research (A*STAR), Singapore; 11 Departments of Physiology & Medicine, National University of Singapore, Singapore

**Keywords:** Biomarker, Brown adipose tissue (BAT), Exosomes, Methylene tetrahydrofolate dehydrogenase 1-like (MTHFD1L), N(10)-formyltetrahydrofolate synthetase

## Abstract

Brown adipose tissue (BAT) is a promising weapon to combat obesity and metabolic disease. BAT is thermogenic and consumes substantial amounts of glucose and fatty acids as fuel for thermogenesis and energy expenditure. To study BAT function in large human longitudinal cohorts, safe and precise detection methodologies are needed. Although regarded a gold standard, the foray of PET-CT into BAT research and clinical applications is limited by its high ionizing radiation doses. Here, we show that brown adipocytes release exosomes in blood plasma that can be utilized to assess BAT activity. In the present study, we investigated circulating protein biomarkers that can accurately and reliably reflect BAT activation triggered by cold exposure, capsinoids ingestion and thyroid hormone excess in humans. We discovered an exosomal protein, methylene tetrahydrofolate dehydrogenase (NADP^+^ dependent) 1-like (MTHFD1L), to be overexpressed and detectable in plasma for all three modes of BAT activation in human subjects. This mitochondrial protein is packaged as a cargo within multivesicular bodies of the endosomal compartment and secreted as exosomes via exocytosis from activated brown adipocytes into the circulation. To support MTHFD1L as a conserved BAT activation response in other vertebrates, we examined a rodent model and also proved its presence in blood of rats following BAT activation by cold exposure. Plasma concentration of exosomal MTHFD1L correlated with human BAT activity as confirmed by PET-MR in humans and supported by data from rats. Thus, we deduce that MTHFD1L appears to be overexpressed in activated BAT compared to BAT in the basal nonstimulated state.

## Introduction

It is well established that brown adipose tissue (BAT) activity in adult humans can be demonstrated via positron emission tomography combined with computed tomography (PET-CT) for over a decade [1]. One could perhaps argue that the initial triumphs back then probably contributed to the long-accepted view of this technique being heralded as the ‘gold standard’ for BAT imaging [2,3]. While this has sparked a new resurgence of interest to explore the feasibility of BAT for the amelioration of obesity and diabetes mellitus that jointly afflict over a billion people globally, this mission is significantly curtailed by the two major drawbacks of PET-CT, namely its high ionizing radiation dose and its exorbitant cost. Because the investigation and monitoring of the effectiveness of any BAT-based treatment modality would require repeated serial PET-CT scans, there is a quest for an alternative safer and cheaper BAT activity detection methodology that will facilitate and accelerate BAT research. Over time, such efforts have led to the emergence of a slew of newer BAT detection strategies that include fat fraction magnetic resonance imaging (MR FF), infrared thermography (IRT), near infrared spectroscopy (NIRS) and indirect calorimetry, all with the hope that a safer and more cost-effective modality with good reliability and precision can one day replace PET-CT as the gold standard [4–14].

One of the most convenient and readily acceptable form of clinical evaluation in medical practice as well as research is a blood test via venipuncture or finger prick. This motivates us to consider elucidating a circulating biomarker that can reliably indicate BAT activation. As one of the more recent paradigms of inter-organ crosstalk involves exosomes (membrane bound extracellular vesicles 30–100 nm in size, produced in the endosomal compartment of most eukaryotic cells) [15], we decided to profile the exosomal proteomics of active BAT. The aim of this present work was to explore the hypothetical assumption that such a unique circulating protein biomarker which accurately reflects BAT activation actually exist. We therefore examined whether such an exosomal protein would be found to be elevated when BAT is activated by known classical stimuli including cold exposure, capsinoids ingestion and thyroid hormone excess in people with hyperthyroidism.

## Materials and methods

### Human subjects

Healthy volunteers aged 21–40 years old with BMI ranging from 18.5 to 29.9 kg/m^2^ were recruited into a study acronymed, TACTICAL-II that investigated BAT activation and energy expenditure elevation stimulated by cold and capsinoids (www.ClinicalTrials.gov identifier NCT02964442) [16]. Subjects were cold exposed for about 2 h by wearing a cooling vest at a constant temperature of 14.5°C (Cool58, Polar Products, Ohio, U.S.A.). On a separate study visit separated at least 48 h apart, subjects were given capsinoids capsules (12 mg, 8 gel capsules) provided by Ajinomoto Inc. (Tokyo, Japan), the composition of which can has been described previously [17]. Active BAT was detected using fusion positron emission tomography with magnetic resonance imaging scanner (PET-MRI). Participants with adipose tissue uptake exhibiting PET SUV mean ≥ 2 were defined to be BAT-positive as described in detail in the literature [9,18,19]. Those below this cutoff were defined as BAT-negative. In total, 12 BAT-positive healthy subjects (6 males/6 females) were identified accordingly. The remaining 8 subjects (2 males/6 females) were BAT-negative. All subjects had measurements of PET SUV and magnetic resonance fat fraction (MR FF) via PET-MRI imaging after cold exposure for around 2 h, infrared thermography maximal temperature (IR Tscv max), energy expenditure by whole body calorimetry (EE) and blood sampling for BAT secretome analysis before and after 2 h of BAT-activating stimulation. Hyperthyroid patients were recruited into a study acronymed, TRIBUTE (www.ClinicalTrials.gov identifier NCT03064542) [20], which investigated BAT activation due to thyroid hormone excess, and followed through from pretreatment till euthyroidism was achieved as proven by standard thyroid function tests based on plasma free thyroxine, free triiodothyronine and TSH levels. The same protocol of PET-SUV, MR FF, IRT Tmax and EE were measured in the hyperthyroid and euthyroid states. Pooled plasma were collected from the three groups (cold, capsinoids, hyperthyroid). For the purpose of selecting the BAT-positive samples in the TACTICAL-II study: *N* = 3–5 subjects’ samples from the strongest PET-SUV response were pooled together to form four respective tubes, each containing at least 5 ml pooled plasma for optimal exosome enrichment and mass spectrometry consisting of 1 tube pre-cold stimulation pooled plasma, 1 tube post-cold stimulation pooled plasma, 1 tube of pre-capsinoids pooled plasma and 1 tube of post-capsinoids pooled plasma. For TRIBUTE samples: *N* = 3–5 patients with the strongest PET SUV were pooled to get 1 tube of 5 ml pooled plasma taken when they were pre-treated in the hyperthyroid state and 1 tube of 5 ml pooled plasma after the same patients were rendered euthyroid with carbimazole. Both studies were conducted according to the ethical guidelines of the Declaration of Helsinki, and all procedures were approved by the Domain-Specific Review Board of National Healthcare Group, Singapore (IRB codes: C/2015/00715 and C/2015/00718). Written and witnessed informed consent was obtained from all subjects before their formal participation.

### Human exosome isolation and tandem mass spectrometry

Exosome analysis was performed via a well-established proteomic workflow. Separation of extracellular vesicles from human plasma was performed by differential centrifugation [21,22]. Frozen individual plasma samples were thawed on ice and pooled in a group-wise manner as described above to obtain tubes containing around 5 ml of plasma specimens per group. The samples underwent sequential centrifugation to enrich the extracellular vesicles including microvesicles and exosomes using a modified protocol as previously described [23,24]. The purified exosomes were dissolved in TRIZOL buffer for protein isolation and purification. Exosomal proteome was profiled using Tandem Mass Tag (TMT) method coupled with Multi-Dimensional Protein Identification Technology (MuDPIT) [25]. Each sample was run in three times. The labeled exosomal proteins with TMT tags were fractioned by HpHRP liquid chromatograph and analyzed by hybrid Quadrupole-Orbitrap LC-MS/MS using Q-Exactive LC-MS/MS system. The raw data generated by Q-Exactive LC-MS/MS was analyzed by ProteomeDiscoverer with Sequest HT and Mascot software (Thermo-Fisher, San Jose, CA). The initial dataset was filtered through stringent elimination criteria of <1% false discovery rate (FDR) to obtain confident identification, CV < 10% and *P-*value <0.05 to get rid of the technical variations during quantization. Stringent statistical analysis such as volcano plot was used to determine the significant cut-off value of fold-change. Proteins with significant fold-changes that passed the stringent filtering criteria were then taken for further data mining and bioinformatics analysis such as gene ontology and pathway analysis. Potential candidate biomarkers were shortlisted from the significant expression changed proteins that are in critical nodes or switches of obesity pathology or WAT/BAT physiology. The mass spectrometry proteomics data have been deposited to the ProteomeXchange Consortium via the PRIDE [26] partner repository with the dataset identifier PXD023909.

### Animals

All experimental procedures and animal research were approved by the institutional animal care and use committee (IACUC, IACUC code number 181361). Animals were acquired from InVivos, Singapore and housed in designated holding room controlled for a 12-h light, 12-h dark cycle, temperature, humidity and quality of the air. All the animal experiment conducted at Biological Resource Centre is an AAALAC-accredited facility located in the Biopolis campus, Singapore. Male Wistar rats (InVivos, Singapore) at 7 weeks of age were randomized into two groups and assigned as thermoneutral (*n*=5) and cold exposed (*n*=5). Both the groups of animals were maintained on chow diet (CD) for further 8 weeks (indicated as 15 weeks of age). The diet composition of the CD (Altromin #1324 mod, Altromin, Germany) includes (6% fat and 18% protein) essential vitamins and proteins.

### Cold exposure in rats

The animals from thermoneutral group were maintained at 27–28°C. Prior to exposing the animals to 4°C, the animals were habituated to cold temperature to stabilize the metabolic alterations. The animals in the cold exposure group were subjected to cold exposure at 18°C for 3 days, 10°C for 3 days and 4°C for 1 day before the experiments. After cold exposure habituation, these animals were then exposed to 4°C for 2 weeks. The health condition of animals were monitored during entire period. All animals survived the cold exposure intervention. The protocol had been approved by the institutional animal ethics board (IACUC code number 181361) prior to the conduct of the study.

### Blood and tissue collection in rats

Experiment rats were killed by increasing concentration of CO_2_ 70% in the enthanizing chamber for about 3–5 min. Blood was collected from the heart by cardiac puncture from the euthanized animal using 5 ml syringe with a 23G needle in sterile 2 ml Eppendorf tubes containing 10–30 USP units of heparin/ml of blood. Blood samples were kept on ice and centrifuged at 12000 rpm for 10 min to collect the plasma. Plasma samples were aliquoted and stored at −80°C for further experiments. Brown adipose tissues were harvested from interscapular region and snap frozen with liquid nitrogen and stored at −80°C for further analysis.

### Plasma exosome isolation in rats

Isolation of exosomes from plasma was performed by using total exosome isolation kit (Thermo Fisher Scientific, catalogue # 4484450). The isolation was performed according to the protocol of the supplier. Plasma sample of 100 μl was used for the analysis and samples were centrifuged (Eppendorf centrifuge 5417R). Exosome pellets (kit isolates) were suspended in exosome resuspension buffer provided by supplier.

### Exosome protein isolation in rats

Isolation of protein from exosome was performed with the total exosome RNA & protein isolation kit (Fisher Scientific, catalogue # 4478545). Isolation of the protein was performed according to the protocol of the supplier. About 5 μl of each samples were loaded for Western blot experiment.

### Western blot for exosomal proteins in rats

Isolated exosome protein pellets were resuspended in 30 μl of exosome resuspension buffer provided by supplier. Equal amounts of protein (5 μl) were separated using 4–12% NuPage mini precast gels (Invitrogen Inc.) followed by dry transfer for Western blot experiments using the iBlot®2 Dry Blotting with Blot®2 NC regular Stacks. After transferring from dry blot, nitrocellulose membranes were stained with Ponceau S and images were acquired. Membranes were then blocked with 5% BSA in TBST for 1 h at room temperature. Overnight incubation of membranes at 4°C with primary antibodies for Anti-MTHFD1L antibody+5% BSA (ab116615, Abcam, Cambridge, MA, U.S.A.) and washed with TBST 5 min for 5 times, followed by 1 h exposure at room temperature with fluorescently-labeled secondary antibody 5% BSA buffer (IRDye® 800CW Donkey anti-Goat IgG); 1:10,000; cat. no. 925-32214 and washed with TBST 5 min for 5 times. Membrane scanned using an Odyssey Infrared Imaging system with IR fluorescence scan Odyssey 2.1 software (LI-COR Biotechnology).

### Real-time PCR for rat samples

Total RNA was isolated from the intrascapdular brown adipose tissue using RNeasy Lipid Tissue Mini Kit (Qiagen 74804) and cDNA conversion using a revertAid H minus first strand cDNA synthesis kit (Thermo Scientific k1632) with oligo d(T) 18 primer according to the manufacturer’s instructions. Real-time qPCR, cDNA samples were analyzed in duplicate using the SYBR Green PCR Master Mix reagent kit (Applied Biosystems 4367659) on a StepOnePlus Real-Time PCR System (Applied Biosystems). Relative mRNA levels were calculated and normalized to 36B4 Forward (TTCCCACTGGCTGAAAAGGT) and (GCCGCAGCCGCAAATGC), used as an endogenous control gene. The primer sequences used for the Methylenetetrahydrofolate Dehydrogenase (NADP+ Dependent) 1-Like were as follows: MTHFD1L (TGCCGAGGGACTTCATTCTG) and (ACCTGGCATTGTGCTCATCA). Fold change in mRNA expression was calculated by *delta*-*delta* Ct method. Statistical significance was assessed by two-tailed Welch’s *t* tests to analyze the differences between two groups and statistical significance was defined as *P*<0.05.

### Data analysis

For rat samples, Western blot data were analyzed using Odyssey software as per manufactures guidelines. Ponceau intensity was analysed using ImageJ software. The average values were expressed as mean ± SD. Statistical significance was assessed by two-tailed Welch’s *t* tests to analyze the differences between the two groups and statistical significance defined as *P*<0.05. For the human data, these are presented as mean ± standard deviation (SD) unless otherwise stated. A *P*-value < 0.05 was considered statistically significant. Statistical analysis was performed in Stata MP V14.0 (Stata Corp, Texas, U.S.A.) for the confirmatory analysis and other statistical analysis was performed in SPSS software version 23 (IBM SPSS Inc.).

### Statistics and bioinformatics analysis

Only proteins that were identified in at least two technical replicates from controls and at least two technical replicates from cases (defined as the group where we anticipate higher BAT activity) were used for statistical analysis. The mass-by-charge (*M/Z*) abundance data was scaled within for each sample. The fold-change between cases and controls was calculated as the ratio of the group means. The difference in the group means was tested using a two sample *t*-test. The corresponding p-value was adjusted for multiple hypothesis testing using the FDR [27]. Proteins with FDR < 5% and at least 1.2-fold change in the capsinoid stimulation study was considered significant. Similarly, FDR < 5% and 1.3-fold change was used for the cold exposure stimulation study and FDR < 5% and 1.5-fold change for hyperthyroid treatment study. Volcano plots were generated using the Enhanced Volcano plot package [28]. The *P*-value corresponding to FDR of 5% and the study specific fold-change cut-offs are represented as horizontal and vertical lines. All statistical analysis was carried out using R package version 3.6.0. [29].

## Results

### Profiling the activated BAT proteome to identify a unique signature or protein that characterizes BAT activation

**Figure 1:**
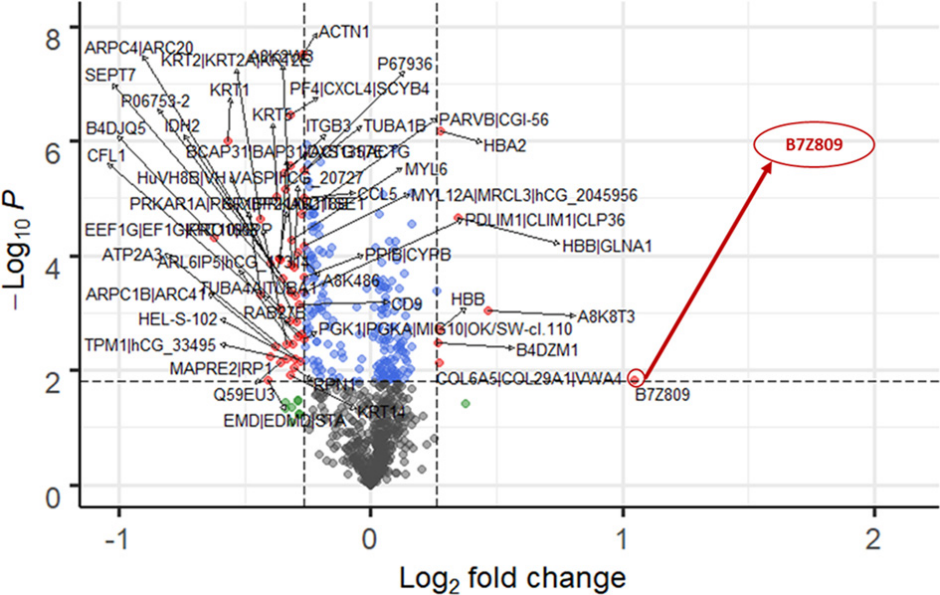
BAT activated protein expression following acute cold stimulation Volcano plot showing protein expression levels in pooled plasma samples of human subjects (*N*=5) under acute cold stimulation with resultant BAT activation. Among the upregulated proteins, ‘highly similar to C-1-tetrahydrofolate synthase (B7Z809)’ was significantly increased. FDR < 0.05 and FC cutoff = log_2_ (1.2).

**Figure 2:**
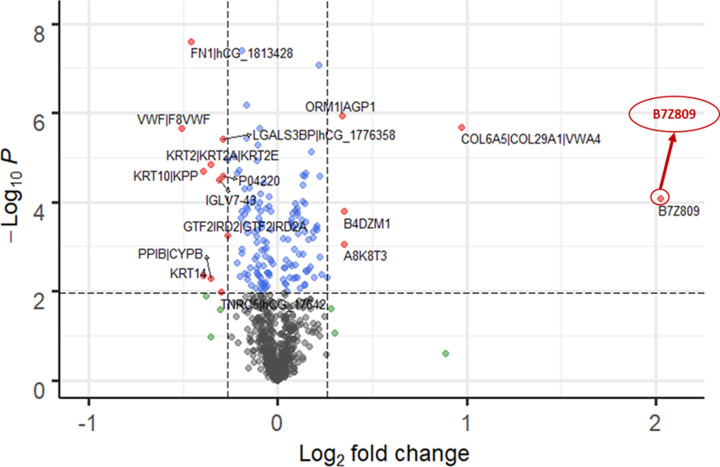
BAT activated protein expression following acute capsinoid stimulation Volcano plot showing protein expression levels in pooled plasma samples of human subjects (*N*=5) under acute capsinoids stimulation with resultant BAT activation. Among the upregulated proteins, ‘highly similar to C-1-tetrahydrofolate synthase (B7Z809)’ was significantly increased. FDR < 0.05 and FC cutoff = log_2_ (1.2).

**Figure 3:**
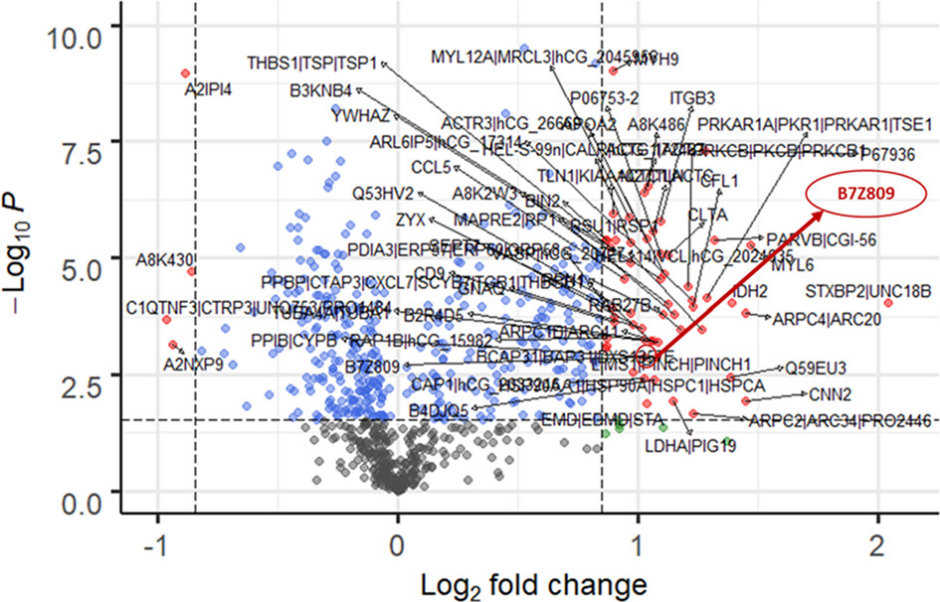
BAT activated protein expression in hyperthyroid patients Volcano plot showing protein expression levels in pooled plasma samples of hyperthyroid patients (*N*=3) with BAT activation. Among the up-regulated proteins, ‘highly similar to C-1-tetrahydrofolate synthase (B7Z809)’ was significantly increased. FDR < 0.05 and FC cutoff = log_2_ (1.2).

In our quest to unravel the BAT secretome, we analyzed the detailed proteomic dataset generated from mass spectrometry to discover the existence of novel exosomal-based biomarkers. Using a standardized bioinformatics approach, we represented the data of the BAT secreted proteome in the cold-stimulated, capsinoid-stimulated and hyperthyroid patients in the form of three volcano plots (Figures 1-3). In regard to the protein expression levels in pooled plasma of human subjects under acute cold stimulation with resultant BAT activation, 9 exosomal proteins shows unique expression with acute cold stimulation. Among the up-regulated and down-regulated exosomal proteins, 3 were up-regulated and 6 were down-regulated significantly. For the subjects under acute capsinoids stimulation with resultant BAT activation, 8 exosomal proteins show unique expression under capsinoids stimulation. Among the up-regulated and down-regulated exosomal proteins, 1 were up-regulated and 7 were down-regulated significantly. As for hyperthyroid patients with resultant BAT activation, 97 exosomal proteins show expression unique to the hyperthyroid patients. Among the up-regulated and down-regulated exosomal proteins, 81 were up-regulated and 16 were down-regulated significantly.

Using recommended statistical cutoffs, a common exosomal protein, B7Z809 (UniProtKB taxonomy symbol for ‘highly similar to C-1-tetrahydrofolate synthase, *Homo sapiens*’), was found to be present in the plasma following cold-stimulation and capsinoids ingestion in healthy lean human subjects as confirmed BAT-positive by PET-MRI. We also found this same protein to be overexpressed among hyperthyroid patients whose BAT was activated by thyroid hormones. Notably, the thyroid hormone, triiodothyronine (T3), is known to be a critical factor required for brown adipocyte differentiation as well as activation [30]. Hence, it is likely to have activated BAT among those with thyroid hormone excess, a state which could partly explain the rapid weight loss despite good appetite in these cases due to fat catabolism by activated BAT. As B7Z809 is the UniProtKB taxonomy symbol for the protein, ‘highly similar to C-1-(methylene)-tetrahydrofolate synthase, *Homo sapiens*’, we deduced that the enzyme, methylene tetrahydrofolate dehydrogenase 1-like (MTHFD1L), is the protein that is over-expressed by activated BAT. This is because MTHFD1L is a mitochondrial enzyme, and BAT which is richly endowed with mitochondria can conceivably release it during activation under the appropriate stimuli. Among the upregulated proteins, B7Z809 was significantly increased (FDR < 0.05 and FC cutoff = log_2_ (1.2)) with all the three modes of BAT activation.

### Proteome correlation differs by method of BAT activation

**Figure 4:**
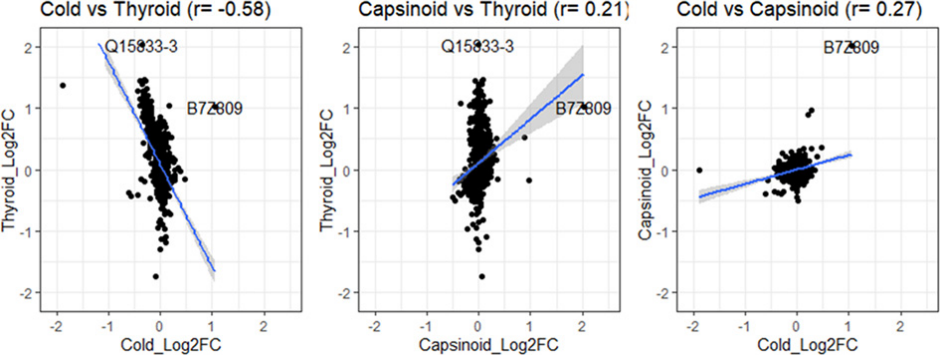
Correlation patterns of exosomal protein expressions for cold versus capsinoid versus hyperthyroid states of BAT activation The pattern of protein expression in the pooled plasma samples appears to be similar between cold-stimulated (*N*=5) and capsinoid-stimulated (*N*=5) BAT (**C**), as well as between capsinoid-stimulated (*N*=5) and hyperthyroid-stimulated (*N*=3) BAT (**B**). However, the protein expression profile for hyperthyroid-stimulated and cold-stimulated BAT (**A**) appears to be inversely correlated.

Despite having found B7Z809 as a unique protein overexpressed in cold-stimulated, capsinoid-stimulated and thyroid hormone-stimulated BAT, it is insightful to determine if other proteins secreted by activated BAT are also similar of different based on the method of BAT activation. We therefore correlated the proteome signatures of these differently activated BAT against each other. Enigmatically, the secreted proteome of BAT activated by the hyperthyroid state varied inversely with that by cold stimulation (Figure 4A). However, the secreted proteome of capsinoids-activated BAT correlated to the secreted proteome of cold-activated BAT and also BAT activated by hyperthyroidism (Figure 4B,C).

### B7Z809 is the common exosomal protein of BAT activation

**Figure 5:**
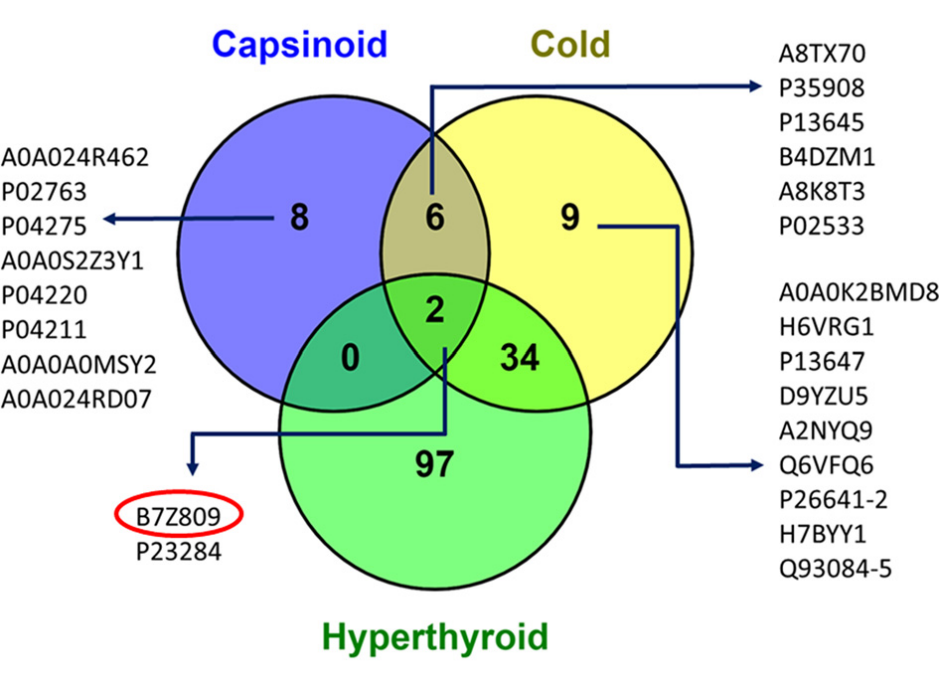
VENN diagram of common and uniquely expressed genes across the three different modes of BAT activation (*N*=13 in total, analyzed by pooled samples)

Using a Venn diagram representation, we overlapped all the secreted proteome of BAT activated by all three modes of stimulation and this revealed two proteins at the intersection of the three groups, namely B7Z809 (highly similar to C-1-tetrahydrofolate synthase, *Homo sapiens*) and P23284 (PPIB). As *PPIB* (peptidylpropyl isomerase B) encodes for cyclophilin B protein which has a stable expression in peripheral whole blood and used to be an internal standard for whole blood mRNA quantification assays, this implied that the only protein that is overexpressed when BAT is activated is B7Z809 (Figure 5). As B7Z809 is an enzyme with C-1-tetrahydrofolate synthase activity, this matches mitochondrial monofunctional C-1-tetrahydrofolate dehydrogenase-1-like (MTHFD1L) perfectly.

### MTHFD1L protein is also overexpressed in activated BAT of rodents

**Figure 6:**
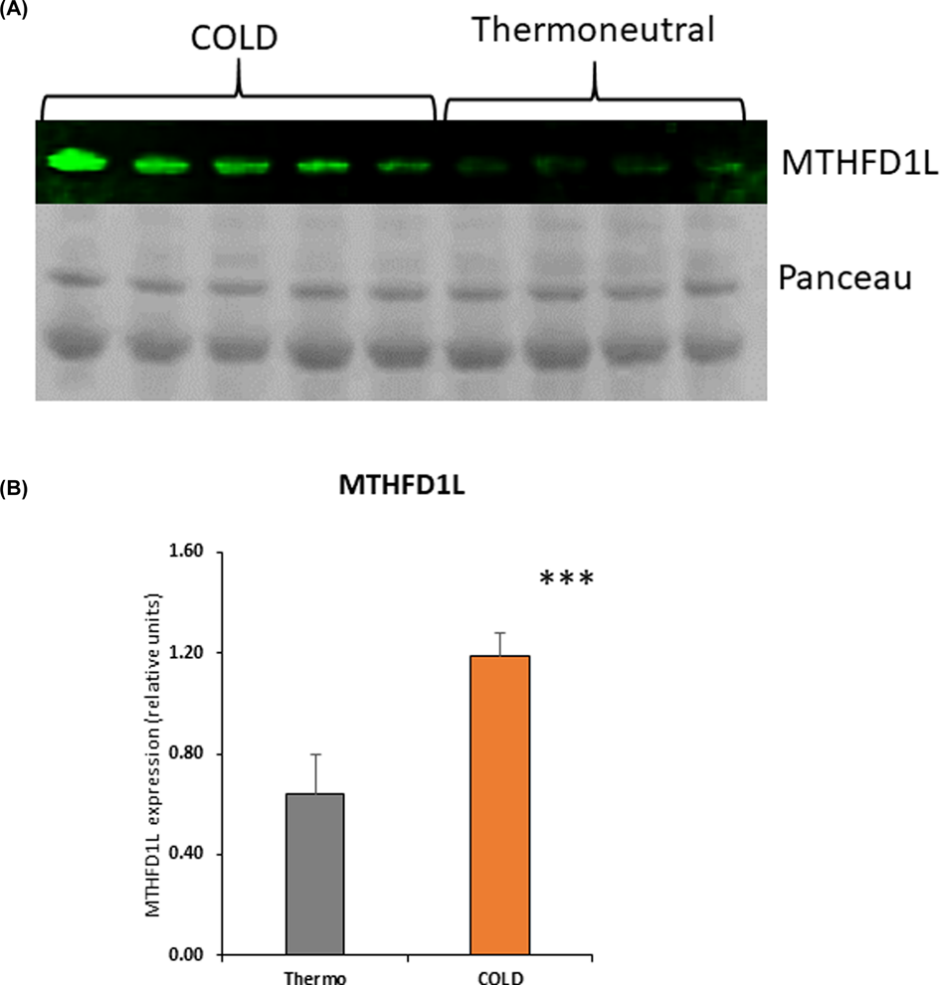
Western blot analysis of MTHFD1L protein isolated from rat plasma (**A**) Western blot images showing upregulated expression MTHFD1L protein in the circulation after 2 weeks of cold exposure at 4°C compared with basal expression in the thermoneutral group (**B**) Western blot densitometry analysis showing significant increase of MTHFD1L in plasma following cold exposure compared to the thermoneutral group (*** *P*<0.001).

To further explore if MTHFD1L is a protein secreted by activated BAT into the circulation in other vertebrates, we examined the exosomes in plasma of rats exposed to cold. Using Western blots, we confirmed that MTHFD1L isolated from rat plasma was significantly higher in rats exposed to 2 weeks of cold at 4°C compared with the basal levels under thermoneutral condition prior to cold exposure (Figure 6A,B).

### MTHFD1L mRNA is increased in BAT of rats exposed to cold

**Figure 7:**
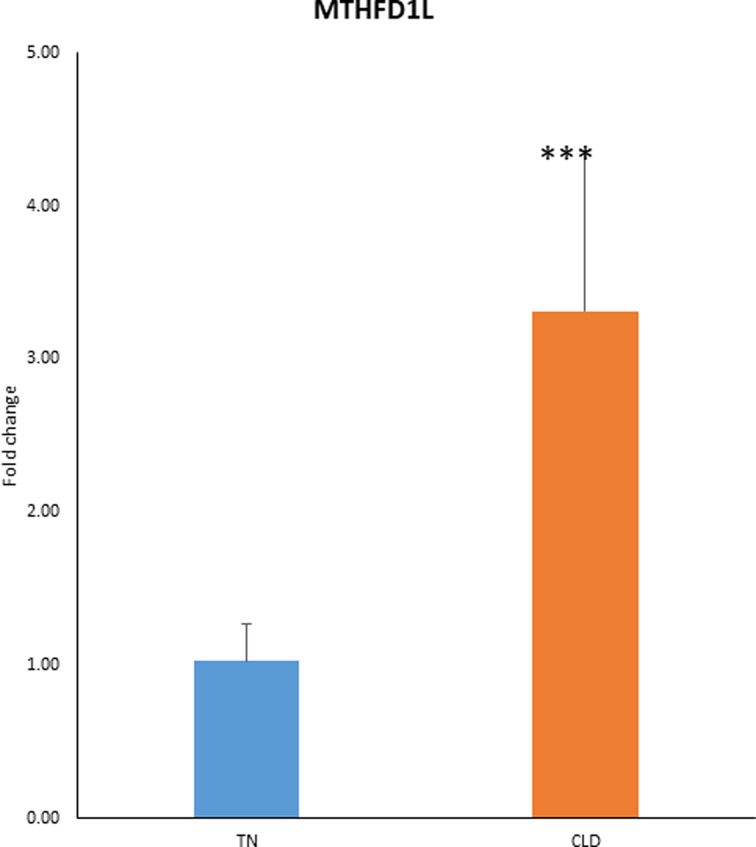
QPCR analysis of mRNA MTHFD1L expression from iBAT mRNA expression of MTHFD1L from intrascapular brown adipose tissue obtained from animals exposed to 2 weeks of cold (4°C) (*N*=5) and thermoneutral condition (*N*=5). There was a significant increase of MTHFD1L in the cold exposed state compared to the group under thermoneutral condition (*** P<0.001).

To demonstrate that MTHFD1L protein is a biomarker of BAT activation, we needed to show that MTHFD1L mRNA is increased in BAT itself. Analysis of the cold-exposed rats revealed that MTHFD1L mRNA from intrascapular brown adipose tissue obtained from animals exposed to 2 weeks of cold (4°C) was 3-fold higher in cold-exposed state compared with the thermoneutral state (Figure 7). These data help to support that the tissue source of overexpression of MTHFD1L arose from the activated BAT.

## Discussion

Over the last decade, BAT has garnered great interest as a therapeutic target to combat obesity and type 2 diabetes as numerous studies have established an association between BAT activity and metabolic health [31–34]. In addition to its metabolic function, BAT is also a source of signaling molecules that can modulate BAT function or even other distant tissues like the liver [35,36]. Based on recent research from other groups having described exosomes being secreted by active BAT [37], we examined our own mass spectrometry dataset of the human plasma samples to determine if any unique proteins of BAT activation could be present. In the present study, we demonstrate the existence of a common exosomal protein, MTHFD1L (Figure 7) that was overexpressed in all three different modes of BAT activation, thereby implying that this circulating exosomal protein may serve as a unique plasma biomarker for BAT activity in rats and humans. The pattern of secreted BAT proteome appears to be similar for capsinoids and cold stimulation methods, as well as between capsinoids and hyperthyroidism. This implies that there are probably commonalities in the genes that are actively transcribed during BAT activation by these different modes of stimulation. But the exosomal proteome of BAT stimulated by cold and by the hyperthyroid state seems to correlate inversely with each other. This is presently an unresolved question and is best addressed by future research. Importantly, we take note that the key purpose of BAT activation by cold is a survival thermogenic response meant to defend the body from hypothermia. On the other hand, in hyperthyroid patients whose BAT is activated by thyroid hormones, these patients are not exposed to the cold. Quite the contrary, hyperthyroid patients often experience heat intolerance with a preference for cold weather [38] and also exhibit hyperhidrosis as a thermoregulatory response instead [38]. Hence, the diametrically opposite situation with respect to thermoregulation could potentially lead to BAT proteomes in cold-stimulated and hyperthyroid-stimulated BAT to correlate inversely. It is possible that the mechanisms and gene programs that are activated in these two methods of BAT stimulation differs in the upstream pathways, but that these pathways somehow converged downstream such that the final end result was BAT activation that led to increased thermogenesis.

Exosomes are found in various body fluids including blood and play a role in exchanging information between cells and tissues [39]. Classically, communication between tissues and organs occur by means of circulating hormones and cytokines as encountered in endocrinology and immunology, and via neural transmission as well described in neurology. Exosomes represent a novel paradigm for such inter-organ cross-talk. Several studies have shown that exosomes can regulate the function of the recipient/target cell and miRNAs are considered as major exosomal signals and effectors [39]. For example, exosomes play a pivotal role in adipose *Sirt1* deficiency-mediated obesity and insulin resistance [40]. In the present study, we find a range of secreted exosomal proteins in all three modes of BAT activation. Importantly, we are the first to show that exosomal MTHFD1L is a common protein overexpressed in all the three different modes of BAT activation in humans. The large repertoire of the activated BAT secretome include exosomal cargoes of peptides, proteins and even nucleic acids such as ncRNA (eg. miRNA) from BAT that are likely to exert certain effects on other target tissues of the body with various physiological consequences. For instance, our group had demonstrated that lncRNA is intimately linked to BAT physiology [41,42]. It would require further studies to elucidate the functions of the BAT proteome.

MTHFD1L is methylenetetrahydrofolate dehydrogenase 1–like protein, a mitochondrial monofunctional enzyme encoded by the *MTHFD1L* gene localized on chromosome 6q25.1 [43] with N(10)-formyltetrahydrofolate synthetase activity. This catalyzes the synthesis of tetrahydrofolate (THF) in the mitochondria, a crucial step necessary for the *de novo* synthesis of purines and thymidylate and hence, mitochondrial mtDNA [44]. N(10)-formyltetrahydrofolate is also required for the formation of the mitochondrial initiator methionine tRNAs to drive the translation of mitochondrially encoded proteins [45]. Presumably, when BAT becomes activated, it leads to a cascade of events that orchestrate mitochondrial fusion–fission dynamics to adapt to the heightened catabolic thermogenic state [46]. MTHFD1L is translocated into the mitochondria soon after nuclear transit of MTHFD1L mRNA into the cytoplasm for translation at the rough endoplasmic reticulum and is very likely critical for BAT activity. The newly synthesized MTHFD1L preprotein in the cytosol is kept soluble by molecular chaperones such as heat shock protein-90 or heat shock cognate-70 (Hsp90 or Hsc70) which then guide it through the mitochondria where it interacts with the mitochondrial outer membrane translocase (Tom) complex, the main protein gateway of the mitochondria, with Tom40-formed channel being the key portal for MTHFD1L entry [47–49]. The translocase of the inner membrane (Tim) then import the preprotein to the mitochondrial inner membrane where MTHFD1L is shown to be tightly associated with the matrix side of the mitochondrial inner membrane [50]. Our data supported that this mitochondrial enzyme, MTHFD1L, is the common protein released into the bloodstream upon activation of BAT. The actual molecular mechanisms of the release of MTHFD1L into the circulation is however presently unresolved. While being speculative, MTHFD1L might find its way out from the mitochondria to the cytoplasm during the dynamic processes of mitochondrial fission-fusion with changes in BAT activity and leaks out of active brown adipocytes to the surrounding dense capillary network richly supplying brown fat tissues. Alternatively, because MTHFD1L is encoded by the nuclear chromosomes and not by mitochondrial DNA, it is also possible that the heightened rate of *MTHFD1L* gene transcription and subsequent mRNA translation in the ribosomes of the rough endoplasmic reticulum within the cytoplasm could result in some MTHFD1L protein being channeled into endosomes fated for the exosomal secretory pathway instead of being fully shuttled into the mitochondria of active brown adipocytes.

The demonstration of MTHFD1L overexpression in rats exposed to cold showed that this protein is not merely a specific finding confined to humans but potentially a conserved BAT secretome response in other vertebrates as well. It is therefore useful to investigate if the over-expression of MTHFD1L is also applicable to other BAT-possessing non-human mammals surviving and/or hibernating in cold wintry climates.

The detection of BAT has relied mainly on PET-CT imaging of ^18^F-FDG uptake into metabolically active BAT for over a decade. This imaging technique was originally developed—and is still mainly used for detection of metastasis in oncology. However, PET-CT suffers from a major drawback of exposing people to large doses of ionizing radiation contributed by both the ^18^F-FDG tracer and the CT scan, and thus cannot be used for screening purposes or in the longitudinal study of a large number of healthy subjects in a population. Thus, novel diagnostic biomarkers of BAT activity that are safe, highly repeatable and of low cost and will be important to help accelerate BAT research in order to better understand the physiological role of human BAT in health and disease, as well as for clinical trials to stratify subjects and to quantify the effects of drug candidates on BAT. In this regard, the exosomal protein, MTHFD1L, in human and rat blood samples is highly promising. However, the correlation is modest and future studies with larger cohorts are needed, since biomarkers typically need to classify with great accuracy.

Our study has several strengths. First, the use of PET-MRI for unequivocally defining BAT-positive individuals allow the described findings to be directly attributable to BAT activation. Next, we utilized a powerful method of mass spectrometry for proteomic analysis combined with sophisticated bioinformatics which enabled the identification of the activated BAT secretome. Finally, the use of a rodent model added further support to the human findings and provided a layer of evidence and insight suggesting that the secretion of MTHFD1L by activated BAT is probably not restricted to humans but represents a general response in BAT biology. The weaknesses of our study include the following. The limited aliquots of human plasma exosome samples only permitted pooled plasma analyses and prevented additional protein analysis via Western blots and ELISA as well as RT-qPCR on mRNA to demonstrate that B7Z809 as identified by mass spectrometry corresponded to MTHFD1L. Nevertheless, the mass spectrometry mass-to-charge (*M/Z*) ratio of B7Z809 as determined by the system was consistent with MTHFD1L as per bioinformatics classification. Moreover, by our use of an appropriate model organism for BAT activation study (Wistar rats), we unequivocally showed that MTHFD1L was the mitochondrial protein proven to be secreted into plasma via exosomes when BAT is activated. This strongly supports that MTHFD1L is the protein over-expressed by activated BAT in human beings.

## Conclusion

Taken together, MTHFD1L appears overexpressed in activated BAT. This warrants further studies to determine its applicability as a brown fat biomarker for basic science and clinical applications such as the development of a point-of-care test-kit for BAT activity detection in large cohorts of patients. If successful, this can catalyze BAT research and assist with clinical correlation of BAT activity in metabolic disorders such as thyroid dysfunction, obesity and diabetes where BAT may play an important role [51].

## Data Availability

The mass spectrometry proteomics data have been deposited to the ProteomicXchange Consortium via the PRIDE partner repository [25] with the dataset identifier PXD023909.

## Abbreviations

BAT: brown adipose tissue
FDR: false discovery rate
IRT: infrared thermography
MR FF: fat fraction magnetic resonance imaging
MTHFD1L: methylene tetrahydrofolate dehydrogenase (NADP^+^ dependent) 1-like protein
NIRS: near infrared spectroscopy
PET-CT: positron emission tomography combined with computed tomography
THF: tetrahydrofolate
T3: triiodothyronine
